# Molecular Networks Involved in the Immune Control of BK Polyomavirus

**DOI:** 10.1155/2012/972102

**Published:** 2012-12-05

**Authors:** Eva Girmanova, Irena Brabcova, Jiri Klema, Petra Hribova, Mariana Wohlfartova, Jelena Skibova, Ondrej Viklicky

**Affiliations:** ^1^Transplant Laboratory, Institute for Clinical and Experimental Medicine, Videnska 1958, 140 21 Prague 4, Czech Republic; ^2^Faculty of Electrical Engineering, Czech Technical University in Prague, Karlovo namesti 13, 121 35 Prague 2, Czech Republic; ^3^Department of Nephrology, Institute for Clinical and Experimental Medicine, Videnska 1958, 140 21 Prague 4, Czech Republic; ^4^Department of Statistics, Institute for Clinical and Experimental Medicine, Videnska 1958, 140 21 Prague 4, Czech Republic

## Abstract

BK polyomavirus infection is the important cause of virus-related nephropathy following kidney transplantation. BK virus reactivates in 30%–80% of kidney transplant recipients resulting in BK virus-related nephropathy in 1%–10% of cases. Currently, the molecular processes associated with asymptomatic infections in transplant patients infected with BK virus remain unclear. In this study we evaluate intrarenal molecular processes during different stages of BKV infection. The gene expression profiles of 90 target genes known to be associated with immune response were evaluated in kidney graft biopsy material using TaqMan low density array. Three patient groups were examined: control patients with no evidence of BK virus reactivation (*n* = 11), infected asymptomatic patients (*n* = 9), and patients with BK virus nephropathy (*n* = 10). Analysis of biopsies from asymptomatic viruria patients resulted in the identification of 5 differentially expressed genes (*CD3E*, *CD68*, *CCR2*, *ICAM-1*, and *SKI*) (*P* < 0.05), and functional analysis showed a significantly heightened presence of costimulatory signals (e.g., *CD40/CD40L*; *P* < 0.05). Gene ontology analysis revealed several biological networks associated with BKV immune control in comparison to the control group. This study demonstrated that asymptomatic BK viruria is associated with a different intrarenal regulation of several genes implicating in antiviral immune response.

## 1. Introduction

Innovations to immunosuppressive regimens have improved patient and kidney transplant survival rates; however, drug-induced immune suppression has also resulted in significant increases in complications associated with infections. BK polyomavirus (BKV) infections have emerged as an important cause of virus-related nephropathy following kidney transplantation in the era of modern immunosuppressive therapies [1]. BKV has been shown to reactivate in 30%–80% of kidney transplant recipients but only in 1%–10% of cases resulted in the development of BKV nephropathy (BKVN) associated with subsequent kidney graft deterioration and failure [2–5].

Recently, the polymerase chain reaction (PCR) has been used for routine monitoring of BKV replication in peripheral blood [5]. However, PCR screening has demonstrated that a majority of kidney transplant recipients are BKV positive in urine but not blood and never develop BKV nephropathy with graft function deterioration. Furthermore, it was shown that patients with asymptomatic viruria presented with significant BKV viral loads in kidney graft biopsy specimens [6], suggesting successful control of the BKV infection by the host immune system. 

Generally, innate immunity and nonspecific effector mechanisms represent a first line of defense against infection, followed by the development of more specific, acquired immune responses. Active viral replication in tissues has been considered to be a trigger point for inflammation, and cellular immunity has been suggested to play a critical role in viral control. Recently, it was shown that patients with self-limited BKV reactivation without therapeutic intervention developed BKV-specific T cells and cleared BKV rapidly compared to patients suffering from BKV associated-nephropathy [7]. 

However, the process of successful BKV control on molecular level has not been fully described.

In this study, we investigated the intrarenal specific transcripts during a different outcome of BKV infection in kidney graft.

## 2. Materials and Methods

### 2.1. Patients

Based on the BKV monitoring study [8] and histopathological archive of the Institute for Clinical and Experimental Medicine, Prague, different patient groups were established as follows. (i) The asymptomatic viruria group (AV) (*n* = 9) presented with BK viral urine loads higher than 10^7^/mL [9] on the day of protocol biopsy (3 months after transplant). These patients never developed BKV-associated nephropathy and were negative for BKV replication screening in both urine and blood 12 months after transplantation. Protocol kidney graft biopsies revealed normal findings free of rejection and patients had stable graft function. (ii) Control group (NCG) (*n* = 11) consisted of patients who were neither BKV positive in urine nor in serum at any time-point after kidney transplantation. None of the patients exhibited allograft rejection; protocol biopsy was normal and patients had stable f graft function. (iii) The BK virus nephropathy (BKVN) group consisted of patients (*n* = 10) with BKV-associated nephropathy. Eight patients presented with BKV-associated nephropathy prior to the start of the study and archived biopsy specimens for molecular biology were used for analysis. In 2 cases, BKV-associated nephropathy developed during the course of the study. Histological confirmation of BKV-associated nephropathy was defined as detection of viral cytopathic changes with intranuclear inclusion bodies, associated renal tubular epithelial cell injury, including tubular epithelial cell necrosis, and denudation of basement membranes, as well as positive immunohistochemical staining for the SV40 T large antigen. Patients' clinical and demographic variables are to be found in Table 1. 

Three months after transplantation, protocol kidney graft biopsies were performed in all patients from group I and II and a part of the biopsy specimen fixed for molecular biology analysis at a later date. The study protocol was approved by the Ethics Committee of the Institute for Clinical and Experimental Medicine in Prague and a written informed consent was obtained from all patients.

### 2.2. RNA Isolation and TaqMan Low Density Array (TLDA)

Small portions (~2 mm) of the cortical or juxtamedullary zone from biopsy specimens were immediately stored in RNA later (Ambion Corporation, Austin, TX). Renal tissues were homogenized; total RNA were extracted using StrataPrep Total RNA Microprep Kit (Stratagene, La Jolla, CA, USA) and reverse transcribed into complementary DNA (cDNA) as described elsewhere [10].

The gene expression profile of 90 candidate gene targets known to play roles in the elicitation of immune responses (e.g., genes involved in cytokine expression, costimulatory molecules, growth factors, chemokines, immune regulation, apoptosis markers, and ischemia markers) was determined using real-time RT-PCR (2^−ΔΔ*Ct*^) with *GAPDH* as internal control and cDNA from a control kidney serving as the calibrator in 30 renal biopsy specimen analyses. All evaluated genes are described in Table S1 in Supplementary Material available online at doi:10.1155/2012/972102. Each immune TLDA profile contains lyophilized gene expression reagents (primers and probes (FAM labeled)) in a preconfigured 384 well format. Two samples in duplicate were analyzed per card. Each loading port was filled with a 100 *μ*L cDNA, nuclease free water, and 2X TaqMan universal PCR master mix. Following centrifugation, cards were sealed with a TLDA sealer (Applied Biosystems, Foster City, CA) to prevent cross-contamination. RT-PCR amplification was performed using an ABI Prism 7900 H.T. Sequence Detection system (Applied Biosystems). TLDA cards were analyzed as relative quantification (RQ) and RQ manager 1.2. software for automated data analysis was used (Applied Biosystems). 

### 2.3. Statistical and Functional Analyses

Continuous variables were presented as the mean ± SD (standard deviation). Statistical analysis of categorical characteristics was performed using the *χ* ^2^ test, of continuous parametric and nonparametric variables using student's *t*-test and Mann-Whitney *U* tests. The Bonferroni correction was used when appropriate. Supervised hierarchical clustering was performed using the MeV (V.3) software in order to visualize results. Gene expression data were compared using the Mann-Whitney *U* test followed by the Bonferroni correction. 

For functional analysis, large-scale data management was used to identify specific transcript patterns. In the set analysis, genes were grouped into sets determined by their annotation and then compared between defined groups. For the functional analysis, 2 types of set analyses were used. Since a limited number of genes were assessed, the fully coupled flux analysis that reflects the analysis of the pathway fragment was used as the first set analysis [11]. Fully coupled flux represents a gene network that corresponds to a pathway in which non-zero flux for one reaction implies a nonzero flux for a second reaction, and vice versa. This flux represents the strongest qualitative connectivity that can be identified in a network. The genes coupled by their enzymatic fluxes were shown to have similar expression patterns, share transcriptional regulators, and frequently reside in the same operon. The second gene set type analysis included genes sharing a common gene ontology [12]. For the use of functional analysis, gene expression data were log transformed and compared between groups with one-way analysis of variance (ANOVA) followed by the Bonferroni correction to appropriately account for multiple comparisons. Set-level analysis was carried out using XGENE.ORG [13, 14].

## 3. Results

### 3.1. Gene Expression Profiles

We first determined whether the 90 candidate target transcripts identified differed in their levels of expression between the groups. Using these criteria, cohorts of patients with asymptomatic viruria and the negative control group were established; we also examined archived biopsies from patients with histologically proven BKV-associated nephropathy.

**Figure 1:**
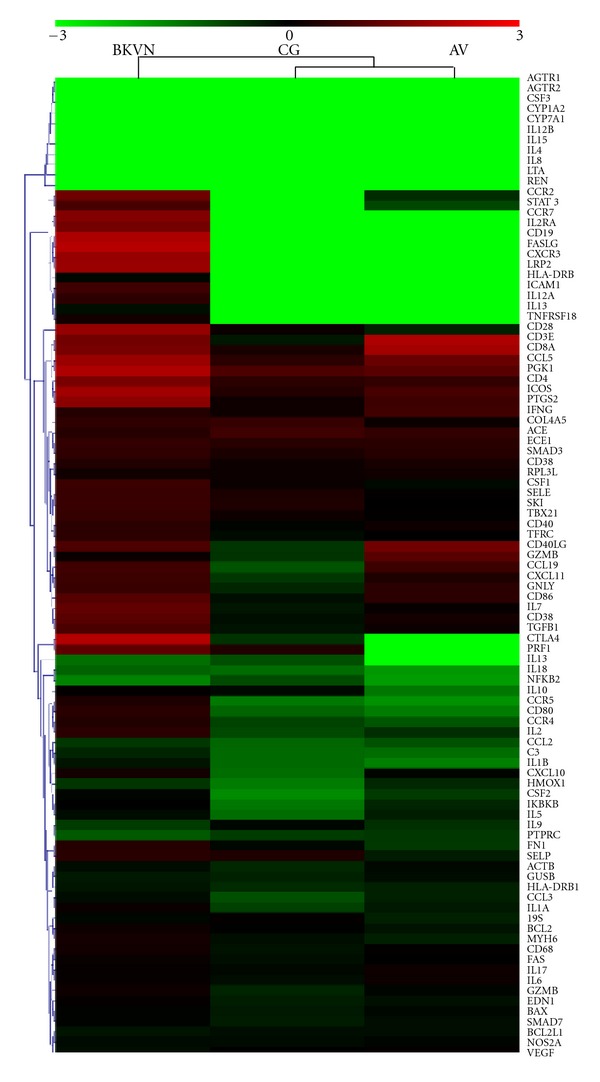
Hierarchical clustering of 3 studied groups. This heat map represents all 90 immune targets assayed (plus endogenous control). Medians of logarithmed relative quantification (RQ) values for each group were used to create the heat map. Red denotes genes with relative increased expression while green denotes genes with relative decreased expression.

Using hierarchical clustering, different gene transcript profiles were identified among the study groups. Hierarchical clustering demonstrated that gene transcript profiles expressed in kidney graft tissues in patients not testing BKV positive were similar to profiles observed in patients presenting with asymptomatic BKV viruria who did not present with BKV positive blood samples or BKV-associated nephropathy. However, gene transcript profiles identified in patients with BKV-associated nephropathy formed a significantly different cluster (Figure 1). 

### 3.2. Characterization of Intrarenal Gene Transcript Profiles in Patients with Asymptomatic BK Viruria

In order to identify the nature of intrarenal immune mechanisms associated with the control of BKV infections, intrarenal graft transcripts from patients with asymptomatic viruria or patients in the negative control group were analyzed. Five differentially expressed genes were identified, including upregulation of the T cell (*CD3E*) and macrophage (*CD68*) markers, in addition to genes encoding the receptor for the monocyte chemoattractant protein-1 (*CCR2*; a chemokine which specifically mediates monocyte chemotaxis) and the adhesion molecule, *ICAM-1*. The *SKI* protooncogene, involved in downregulation of *TGF-*β*1* gene transcripts, was significantly downregulated in the asymptomatic BKV infection group (*P* < 0.05) (Table 2).

Functional analysis revealed that patients with asymptomatic viruria exhibited significantly higher expression levels of the costimulatory signals CD40/CD40L (*P* < 0.05) compared to the negative control group. In addition, groups of genes sharing a common ontology were analyzed, revealing that several biological networks were involved in BKV immune control. These networks were involved primarily with B cell proliferation (*BCL2, CD40, CD40L, IL10*), T cell proliferation (*CD28, CD3E, IL12A, IL4, PTPRC*), transmembrane receptor tyrosine kinase pathways (*CD4, CD8A, FN1*), proteolysis (*ACE, ECE1, GZMB, LTA, REN*), protein kinase binding (*CD3E, CD4, PTPRC*), antiapoptotic processes (*BCL2, BCL2L, CCL2, CD40L, FAS, IL10, IL1A*), and leukocyte adhesion (*CD40L, ICAM1*) (Table 3).

### 3.3. Identification of Intrarenal Gene Transcripts Associated with BKV-Associated Nephropathy

The BKVN group displayed differential regulation of 33/90 genes analyzed. The most differentially regulated genes were *CCL2*, *CCL5*, *CCR2*, *CD4*, *CD68*, *FASL*, *GNLY*, *IL1B*, *IL2RA*, *IL8*, *PRF1*, *PTPRC,* and *TNF* (all *P* < 0.01 compared to the negative control group). These genes are primarily involved in T cell signaling, chemotaxis, activation, and cytotoxicity (Table 4).

Functional analysis revealed that patients with BKVN had significantly higher expression levels of the flux for the *FASL/FAS* (*P* < 0.05) and *CD28*, *CD80*, *CD86* (*P* < 0.05), signaling molecules associated with apoptosis, and costimulation. Moreover, a broad spectrum of molecular networks were shown to have an identical ontology, that is, genes associated with the positive regulation of NF-*κ*B transcription and intracellular signal transduction (*TNF, TGFB1*), chemotaxis (*CCL3, CCL5, IL18, IL1B, IL8, VEGF*), activation of MAPK activity (*IKBKB, TGFB1, TNF*), and negative regulation of viral genome replication (*CD80, IL8*). A detailed description of ontology-related genes associated with BKV-associated nephropathy is listed in Table 5.

## 4. Discussion

Polyoma BK virus-associated nephropathy represents the one of the most challenging infectious complications associated with kidney transplantation [15]. 

A better understanding of the molecular processes associated with the immune control of BKV infections may facilitate improvement of clinical management strategies [16]. Evaluation of BKV in urine samples in this study was performed in a blinded fashion; therefore no changes in clinical management were carried out based on results obtained [8] since it would have influenced the transcript expression profiles present in biopsies collected 3 months after transplantation. To the best of our knowledge, this is the first molecular study in such patient cohort. This analysis demonstrated that effective BKV control 3 months after transplantation was associated with gene expression profiles with the potential of affecting cellular immune responses, including B and T cell signaling and anti-apoptotic gene networks whereas, in late BKV-associated nephropathy, the profound gene upregulation in networks covering T cell signaling, chemoattraction, activation, and cytotoxicity along with many other inflammatory networks were detected.

Specifically, 5 genes likely to be associated with the successful control of viral replication were identified. The heightened presence of T cells (*CD3E*), monocyte macrophages (*CD68*), and their chemoattractant chemokine *CCR2* (as well as presence of adhesion molecule *ICAM-1*) were described. Previous studies have identified these molecules to be associated with viral infections [17, 18]. 

It is broadly known that T cell expansion and cytokine production are needed for the generation of effective antiviral immune responses [19]. CD8^+^ cytotoxic T cells secreting interferon-gamma (IFN-*γ*) or/and tumor necrosis factor alpha (TNF-*α*) are important components in mediating host immunity against viral infections and have been shown to play critical roles in BKV clearance [17]. In our study, however, the expression pattern of *IFN-*γ** and *TNF-*α** during asymptomatic viruria was just marginal (*P* < 0.1) that may reflect just a limited burden of immune injury. CD8^+^ T lymphocyte activation is tightly regulated, especially during primary responses elicited following positive and negative costimulation following BKV infections [20, 21]. In our study, molecules associated with costimulation were consistently upregulated in kidney tissues of asymptomatic viruria patients and also in biopsies from patients with confirmed BKV-associated nephropathy. Therefore, in order to further identify additional genes associated with protection from BKV infection, data from our study was further analyzed by carrying out functional analyses. Since a low number of genes were assessed compared to the number of genes that could be analyzed following a microarray analysis, we focused our research on describing smaller interactional and functional units using a gene ontology approach and network flux that corresponds to a part of the pathway.

Flux for CD40/CD40L costimulatory signal was significantly upregulated in biopsies from patients who successfully controlled BKV infection and presented with asymptomatic viruria. It is well known that activation of CD40 on antigen presenting cells following ligation of CD40L (expressed mainly on CD4^+^ T lymphocytes) contributes to proinflammatory responses necessary for eradication of infections caused by certain types of pathogens [22]. Studies focused on defining cellular immune responses with the potential of controlling BKV replication determined that the majority of the BKV-specific T cells expressed CD40L (CD154) [23].

Gene targets uncovered by gene ontology analysis identified genes typically associated with the elicitation of immune responses specific to viral infections where the interplay between B and T cell function and effective cellular proliferation represents a basic protective strategy. 

The activation of cellular mechanisms in response to BKV infections represents an injury-repair immune response with fibrosis development as a consequence [9]. In the current study, patients with asymptomatic viruria (who never developed BKV-associated nephropathy) presented with normal graft function at the 36-month followup. This meant that successful immune control of viral infection was likely associated with limited or transient cytokine upregulation compared to BKV-associated nephropathy where the burden of injury initiated fibrosis development. 

Currently, there is limited information regarding the molecular processes associated with BKV-associated nephropathy. The upregulation of large number of genes involved in cell cycle and proliferation was shown *in vitro* in BKV infected primary kidney epithelial cells that suggests stimulatory nature of BKV proteins [24]. In vivo, the analysis of genes upregulated in renal allografts affected by BKV-associated nephropathy identified proinflammatory genes (CD8 and related molecules associated with graft fibrosis) similar to the profile observed during cases of acute rejection; however, expression levels were larger in magnitude [25]. In our study, intraparenchymal upregulation of 33 genes was observed in BKV-associated nephropathy, confirming previous results [25], as well as demonstrating a similar expression profile to that observed during acute rejection [25–27]. Moreover, using the functional analysis approach, another 50 biological processes were described in kidneys affected by BKV-associated nephropathy. Using hierarchical clustering, gene expression in BKV-associated nephropathy formed clearly different group.

In this study, the quantitative PCR analysis was performed. Compared to microarray-based analyses, this technique was fast and quantitative, and the results were more reliable. More specific tools for the study of specific immune responses associated with BKV infections would include ELISPOT and multiparameter flow cytometry analyses [7, 17, 28, 29].

## 5. Conclusion

In conclusion, this study demonstrated that asymptomatic BKV viruria reflecting successful immune system control of viral infections was associated with specific gene transcripts and immune processes, specifically transcripts associated with B lymphocyte signaling and costimulation. Furthermore, the degree of associated immune responses was much higher in patients presenting with BKV-associated nephropathy. 

## Supplementary Material

The gene expression profile of 90 candidate gene targets known to play a role in the elicitation of immune responses (e.g., genes involved in cytokine expression, costimulatory molecules, growth factors, chemokines, immune regulation, apoptosis and ischemia markers) was determined using real-time RT-PCR.

## Figures and Tables

**Table 1:** Demographics and transplant related variables.

	CG (*n* = 11)	BKVN (*n* = 10)	AV (*n* = 9)	P* value
At baseline				
Recipient age (years)	50 ± 10	44 ± 21	47 ± 10	ns
Donor age (years)	49.2 ± 13	47 ± 18	46 ± 15	ns
Living donor (*n*, %)	1/9%	3/30%	1/22%	ns
Gender (male, %)	6/54%	3/30%	6/66%	ns
HLA mismatches	3 ± 1	4 ± 1	2 ± 1	ns
Peak level of PRA	5 ± 7	44 ± 40	1 ± 1	ns
CMV serostatus				
*D−/R− *	1/9%	1/10%	1/11%	ns
*D−/R+ *	1/9%	1/10%	1/11%	ns
*D+/R− *	3/27%	1/10%	1/11%	ns
*D+/R+ *	6/54%	7/70%	6/66%	ns
Dialysis before TX (months)	19 ± 10	27 ± 15	32 ± 26	ns

At 3M biopsy				
Time after TX	94.8 ± 5.8	343 ± 268	85.6 ± 13.5	ns
BMI (kg/m^2^)	23.5 ± 4.8	27.3 ± 4.3	27.2 ± 4.7	ns
Immunosuppression				
Induction	36%	50%	44%	ns
Tacrolimus	73%	80%	89%	ns
Cyclosporin A	27%	0%	0	ns
mTOR	18%	40%	11%	ns
Mycophenolate mofetil	55%	70%	89%	ns
Prednisone	73%	90%	89%	ns
CIT (deceased donor)	20 ± 3	13 ± 9	19 ± 3	ns
Serum creatinine (*μ*mol/L)	112 ± 60.3	210 ± 67.2	126 ± 35.3	**
Serum creatinine at 36-month followup	122.6 ± 37.3	NA	144.1 ± 69.5	ns

Data shown as mean ± standard deviation if not indicated otherwise. ESRD: end-stage renal disease; CIT: cold ischemia time; PRA: panel reactive antibody; BMI: body mass index; TX: transplantation; **(*P* < 0.01) BKVN versus negative control group; ns: not significant; *P values for categorical data *χ* ^2^ or Fisherś test and for continuous variables Mann-Whitney *U * test (Student's *t*-test where appropriate).

**Table 2:** Genes with different regulation in asymptomatic viruria group.

Gene	Control group	Asymptomatic viruria group	*P* value*
*CCR2 *	0 (0–6.38)	0.3 (0–31.7)	0.02
*CD3E *	0.53 (0.03–37.3)	117 (0.15–1120)	0.04
*CD68 *	0.61 (0.05–1.38)	1.15 (0.72–4.96)	0.04
*SKI *	2.32 (0.29–13.2)	0.47 (0.07–13)	0.04
*ICAM1 *	0.03 (0–0.08)	0.34 (0.02–2.12)	0.05

Data are shown as median (minimum–maximum) of relative quantity (RQ) of gene expression calculated with regard to the reference gene (*GAPDH*) and calibrator.

*P values calculated by Mann-Whitney *U* test followed by the Bonferroni adjustment.

**Table 3:** Biological networks within graft tissue associated with asymptomatic BK viruria.

GO term		Genes annotated	*P* value*
Soluble fraction	GO:0005625 GO:0005625	*ACTB*, *CCL3*, *CCR2*, *CD40LG*, *FAS*, *IL13*, *SELP *	0.0296
B lymphocyte proliferation, B cell proliferation	GO:0042100	*BCL2*, *CD40*, *CD40LG*, *IL10 *	0.0364
Transmembrane receptor tyrosine kinase pathway	GO:0007169	*CD4*, *CD8A*, *FN1 *	0.0394
Proteolysis	GO:0006508	*ACE*, *ECE1*, *GZMB*, *LTA*, *REN *	0.0416
Protein kinase binding	GO:0019901	*CD3E*, *CD4*, *PTPRC *	0.0426
Platelet activation	GO:0030168	*CD40*, *CD40LG *	0.0428
Positive regulation of T cell proliferation	GO:0042102	*CD28*, *CD3E*, *IL12A*, *IL4*, *PTPRC *	0.0444
Antiapoptosis	GO:0006916	*BCL2*, *BCL2L1*, *CCL2*, *CD40LG*, *FAS*, *IL10*, *IL1A* “*IL1B*,” “*IL2*," “*TNF*,” “*TNFRSF18*”	0.0466
Leukocyte cell-cell adhesion	GO:0007159	*CD40LG*, *ICAM1 *	0.0466
T cell receptor complex	GO:0042101	*CD3E*, *CD4*, *CD8A*	0.0484

*P  values by ANOVA followed by Bonferroni adjustment.

**Table 4:** Genes with different regulation in polyoma BK nephropathy.

Gene	Control group	BKVN group	*P* value*
*C3 *	0.06 (0.02–0.6)	0.375 (0.03–5.27)	0.022
*CCL2 *	0.06 (0.01–0.12)	0.22 (0.03–0.85)	0.005
*CCL3 *	0.12 (0–0.65)	0.71 (0.16–5.89)	0.02
*CCL5 *	3.26 (0.41–45.4)	66.9 (7.9–235)	0.003
*CCR2 *	0 (0–6.38)	20.7 (0–158)	0.001
*CCR7 *	0 (0–659)	35.3 (0–789)	0.017
*CD19 *	0 (0–104)	109 (0–2440)	0.013
*CD28 *	1.36 (0–75.1)	59.3 (0.74–448)	0.012
*CD4 *	3.32 (0.22–15.1)	27 (6.63–209)	0.004
*CD68 *	0.61 (0.05–1.38)	1.68 (0.97–4.79)	0.001
*CD86 *	0.67 (0.01–16.9)	10 (0.88–32.3)	0.012
*CSF1 *	1.42 (0.02–4.89)	5.17 (0.57–71.3)	0.049
*CXCL10 *	0.05 (0–2.9)	1.8 (0.06–3.76)	0.015
*CXCR3 *	0 (0–34.3)	61.2 (0–671)	0.013
*EDN1 *	0.45 (0.14–1.32)	1.18 (0.39–8.74)	0.025
*FASL *	0 (0–67.2)	145.72 (25.96–409.25)	0.003
*GNLY *	0.36 (0.12–2.1)	4.9 (1.45–52.2)	0.001
*HLADRA *	0.39 (0.15–3.27)	1.55 (0.503–7.6)	0.021
*HLA-DRB1 *	0 (0–0.39)	0.85 (0–85.6)	0.036
*ICAM1 *	0.03 (0–0.8)	0.235 (0.06–13.1)	0.03
*IFNG *	2.65 (0–139)	82.1 (0–519)	0.023
*IL12B *	0 (0–3.83)	3.49 (0–37.6)	0.03
*IL1B *	0.17 (0–0.95)	1.38 (0.3–3.67)	0.002
*IL2RA *	0.14 (0–3.44)	3.32 (0.47–19)	0.009
*IL6 *	0.05 (0.01–3.49)	0.72 (0.07–9.49)	0.048
*IL8 *	0.55 (0–6.75)	14.7 (0.11–53.4)	0.004
*LTA *	0 (0–93.9)	68.8 (0–903)	0.036
*PRF1 *	8.7 (0–43.3)	117 (1.4–4100)	0.007
*PTPRC *	1.52 (0.55–24.6)	47.6 (4.7–303)	0.001
*TBX21 *	0 (0–15.2)	7.14 (0–383)	0.036
*TGFB *	0.73 (0.23–6.75)	3.42 (0.73–13)	0.025
*TNF *	0.61 (0.04–3.18)	8.2 (0.95–30.7)	0.002
*TNFRSF18 *	0 (0–1.77)	1.7 (0–19.5)	0.01

Data are shown as median (minimum–maximum) of relative quantity (RQ) of gene expression calculated with regard to the reference gene (*GAPDH*) and calibrator.

*P values calculated by Mann-Whitney *U* test followed by the Bonferroni adjustment.

**Table 5:** Biological networks in kidney graft tissues that are associated with BKVN.

GO term		Genes annotated	*P* value*
Negative regulation of transcription	GO:0048661	*EDN1*, *TNF *	0.003
Positive regulation of NF kappa B transcription	GO:0016481	*TGFB1*, *TNF *	0.004
Activation of MAPK activity	GO:0051092	*IKBKB*, *TGFB1*, *TNF *	0.005
Positive regulation of phosphorylation	GO:0000187	*IL1B*, *TNF *	0.007
Intracellular signal transduction	GO:0001934	*IL1B*, *TNF *	0.007
Negative regulation of viral genome replication	GO:0007242	*CD80*, *IL8 *	0.008
Signal transducer activity	GO:0045071	*CCL5*, *TNF *	0.010
Organ morphogenesis	GO:0004871	*CCL2*, *CCL3*, *CCL5*, *HMOX1*, *IL12B*, *IL13*, *IL15*, *IL18*, *IL1A*, *IL1B*, *STAT3 *	0.011
Positive regulation of transription, DNA dependent	GO:0009887	*CCL2*, *IL7*, *TGFB1*, *TNF *	0.011
Transcription activator activity	GO:0045941	*CD80*, *CD86*, *TNF *	0.011
Exocytosis	GO:0016563	*CD80*, *CD86*, *IKBKB*, *TGFB1 *	0.011
Chemoattractant activity	GO:0006887	*CCL3*, *CCL5 *	0.013
Angiogenesis	GO:0042056	*CCL3*, *CCL5 *	0.013
Induction of positive chemotaxis	GO:0001525	*IL18*, *IL1B*, *IL8*, *VEGF *	0.013
Regulation of cell adhesion	GO:0050930	*IL8*, *VEGF *	0.015
Regulation of isotype switching	GO:0030183	*IL10*, *IL4 *	0.015
Regulation of cell adhesion	GO:0045191	*IL10*, *IL4 *	0.015
Response to oxidative stress	GO:0030155	*ICAM1*, *IL18*, *IL8 *	0.016
Protein phosphorylation	GO:0006979	*CCL5*, *PTGS2 *	0.016
Positive regulation of T helper 2 cell differentiation	GO:0006468	*CCL2*, *IKBKB*, *TGFB1 *	0.017
Cellular component movement	GO:0045630	*CD86*, *IL6 *	0.018
Positive regulation of B cell proliferation	GO:0006928	*ACTB*, *CCL3*, *CCL5*, *CXCR3*, *IFNG*, *IL13*, *IL8*, *PTGS2*, *STAT3 *	0.019
Cellular calcium ion homeostasis	GO:0030890	*IL4*, *IL7*, *PTPRC *	0.019
Chemokine activity	GO:0006874	*CCL19*, *CCL2*, *CCL3*, *CCL5 *	0.022
Response to glucocorticoid stimulus	GO:0008009	*CCL19*, *CCL2*, *CCL3*, *CCL5*, *CXCL10*, *CXCL11*, *IL8 *	0.024
Positive regulationof T cell proliferation	GO:0051384	*IL10*, *IL6*, *TNF *	0.024
T cell differentiation	GO:0042102	*CD28*, *CD3E*, *IL12A*, *IL4*, *PTPRC *	0.024
Defense response to virus	GO:0030217	*IL2*, *PTPRC *	0.025
Induction of apoptosis by extracellular signals	GO:0051607	*BCL2*, *PTPRC *	0.026
Negative regulation of transcription from RNA polymerase II promoter	GO:0008624	*CD38*, *FAS*, *FASLG *	0.027
Positive regulation of protein kinase activity	GO:0000122	*SMAD3*, *STAT3*, *TNF *	0.030
Response to virus	GO:0045860	*CD4*, *PTPRC *	0.033
Negative regulation of cytokine secretion involved in immune response	GO:0009615	*CCL19*, *CCL5*, *IFNG*, *TNF *	0.033
Negative regulation of interleukin-6 production	GO:0002740	*IL10*, *TNF *	0.033
Receptor biosynthetic process	GO:0032715	*IL10*, *TNF *	0.033
Positive regulation of cytokine production	GO:0032800	*IL10*, *TNF *	0.033
Positive regulation of transcription from RNA polymerase II promoter	GO:0050715	*IL10*, *TNF *	0.033
Positive regulation of isotype switching to IgG isotypes	GO:0045944	*IL4*, *IL6*, *SMAD3*, *TNF *	0.034
Regulation of immune response	GO:0048304	*IFNG*, *IL4*, *TBX21 *	0.035
Activation of caspase activity	GO:0050776	*IFNG*, *IL4*, *TBX21 *	0.035
Receptor binding	GO:0006919	*BAX*, *SMAD3*, *TNF *	0.035
Antigen processing and presentation	GO:0005102	*C3*, *REN *	0.038
Activation a proapoptotic gene products	GO:0019882	*CD8A*, *IFNG *	0.038
Cell cycle arrest	GO:0008633	*BCL2*, *FAS*, *FASLG *	0.039
Protein kinase binding	GO:0007050	*IFNG*, *IL12A*, *IL12B*, *IL8*, *SMAD3*, *TGFB1 *	0.042
Cell adhesion	GO:0019901	*CD3E*, *CD4*, *PTPRC *	0.043
JAK-STAT cascade	GO:0007155	*CCL2*, *CCL5*, *CD34*, *CD4*, *CXCR3*, *FN1*, *SELE*, *SELP *	0.044
Plasma membrane	GO:0007259	*CCL2*, *CCR2*, *STAT3 *	0.046
Neutrophil chemotaxis	GO:0005886	*ACE*, *AGTR1*, *AGTR2*, *CCR2*, *CCR4*, *CCR5*, *CCR7*, *CD19*, *CD28*, *CD34*, *CD38*, *CD3E*, *CD4*, *CD40*, *CD40LG*, *CD80*, *CD86*, *CD8A*, *CSF1*, *CXCR3*, *FAS*, *FASLG*, *ICAM1*, *ICOS*, *IL2RA*, *PTPRC*, *SELE*, *SELP*, *TFRC*, *TNF*, *TNFRSF18 *	0.046
Positive regulation of interleukin-2 biosynthetic process	GO:0045086	*IFNG*, *IL1B*, *IL8 *	0.046

*P values by ANOVA followed by Bonferroni adjustment.
