# Supplementary material for: Molecular Networks Involved in the Immune Control of BK Polyomavirus
Source: Clin Dev Immunol. 2012 Dec 5;2012:972102. doi: 10.1155/2012/972102 (PMC3521483; doi:10.1155/2012/972102)
Supplement: Supplementary file 1 — The gene expression profile of 90 candidate gene targets known to play a role in the elicitation of immune responses (e.g., genes involved in cytokine expression, costimulatory molecules, growth factors, chemokines, immune regulation, apoptosis and ischemia markers) was determined using real-time RT-PCR. [file 972102.f1.docx]

| **Supplement Table 1** Genes evaluated by real-time quantitative PCR | | | |
| --- | --- | --- | --- |
| *IL1A* | *CD40* | *GZMB* | *IL9* |
| *18S* | *CD40LG* | *HLA-DRA* | *LRP2* |
| *ACE* | *CD68* | *HLA-DRB1* | *LTA* |
| *ACTB* | *CD80* | *HMOX1* | *MYH6* |
| *AGTR1* | *CD86* | *ICAM1* | *NFKB2* |
| *AGTR2* | *CD8A* | *ICOS* | *NOS2A* |
| *BAX* | *COL4A5* | *IFNG* | *PGK1* |
| *BCL2* | *CSF1* | *IKBKB* | *PRF1* |
| *BCL2L1* | *CSF2* | *IL10* | *PTGS2* |
| *C3* | *CSF3* | *IL12A* | *PTPRC* |
| *CCL19* | *CTLA4* | *IL12B* | *REN* |
| *CCL2* | *CXCL10* | *IL13* | *RPL3L* |
| *CCL3* | *CXCL11* | *IL15* | *SELE* |
| *CCL5* | *CXCR3* | *IL17* | *SELP* |
| *CCR2* | *CYP1A2* | *IL18* | *SKI* |
| *CCR4* | *CYP7A1* | *IL1B* | *SMAD3* |
| *CCR5* | *ECE1* | *IL2* | *SMAD7* |
| *CCR7* | *EDN1* | *IL2RA* | *STAT3* |
| *CD19* | *FAS* | *IL3* | *TBX21* |
| *CD28* | *FASLG* | *IL4* | *TFRC* |
| *CD34* | *FN1* | *IL5* | *TGFB1* |
| *CD38* | *GAPDH* | *IL6* | *TNF* |
| *CD3E* | *GNLY* | *IL7* | *TNFRSF18* |
| *CD4* | *GUSB* | *IL8* | *VEGF* |
